# miR-18a-3p and Its Target Protein HuR May Regulate Myogenic Differentiation in Immune-Mediated Necrotizing Myopathy

**DOI:** 10.3389/fimmu.2021.780237

**Published:** 2022-01-05

**Authors:** Lifang Ye, Yu Zuo, Fang Chen, Qinglin Peng, Xin Lu, Guochun Wang, Xiaoming Shu

**Affiliations:** ^1^ Department of Rheumatology, Key Laboratory of Myositis, China–Japan Friendship Hospital, Beijing, China; ^2^ Peking Union Medical College, Chinese Academy of Medical Sciences, Beijing, China

**Keywords:** HuR, miR-18a-3p, RNA binding protein, immune-mediated necrotizing myopathy, myogenic differentiation

## Abstract

Immune-mediated necrotizing myopathy (IMNM) is characterized by manifestation of myonecrosis and regeneration of muscle fibers; however, the underlying pathogenesis remains unclear. This study aimed to investigate the role and mechanism of miR-18a-3p and its target RNA-binding protein HuR in IMNM. HuR and miR-18a-3p levels were detected in the skeletal muscles of 18 patients with IMNM using quantitative reverse-transcription real-time polymerase chain reaction (qRT-PCR) and western blotting analysis. Human myoblasts were transfected with small interfering RNA targeting HuR and miR-18a-3p mimic or inhibitor. Myogenic differentiation markers, myogenin and myosin heavy chain, were analyzed by qRT-PCR, western blotting analysis, and immunofluorescence staining. The results showed that miR-18a-3p was upregulated (p=0.0002), whereas HuR was downregulated (p=0.002) in the skeletal muscles of patients with IMNM. The expression of miR-18a-3p in patients with IMNM was negatively correlated with those of HuR (r = -0.512, p = 0.029). We also found that disease activity was positively correlated with HuR expression (*r* = 0.576, *p* = 0.012) but muscle activity was negatively correlated with miR-18a-3p expression (*r* = -0.550, *p* = 0.017). Besides, bioinformatics analysis and dual-luciferase reporter assays suggested that miR-18a-3p could directly target HuR. Cellular experiments showed that overexpression of miR-18a-3p inhibited myogenic differentiation by targeting HuR, whereas inhibition of miR-18a-3p led to opposite results. Therefore, miR-18a-3p and its target protein HuR may be responsible for modulating the myogenic process in IMNM and can thus be therapeutic targets for the same.

## Introduction

Immune-mediated necrotizing myopathy (IMNM) is an autoimmune disease that is characterized by severe muscle weakness and myofiber necrosis, but usually low inflammatory cell infiltration and lesser involvement of other organs (1). IMNM consists of three subclasses: anti-signal recognition particle (anti-SRP)-positive, anti-3-hydroxy-3-methylglutaryl-CoA reductase (HMGCR)-positive, and seronegative (2). Myoblast fusion in patients with IMNM is abnormal since the mechanism involved in muscle regeneration is impaired (3); however, the pathogenesis of IMNM is still unclear.

MicroRNAs (miRNA) participate in skeletal muscle differentiation (4). miR-17-92, a highly conserved miRNA cluster, comprises miR-17, miR-20a, miR-18a, miR-19a, miR-19b-1, and miR-92a-1; these miRNAs play an essential role in cell proliferation, differentiation, tumorigenesis, and angiogenesis (5–7). The pathogenic role of miRNAs in many diseases, including autoimmune diseases, has been extensively studied. Previous studies have shown that miRNA expression is abnormal in the peripheral blood mononuclear cells, skeletal muscles, plasma, serum, and lungs of patients with idiopathic inflammatory myopathy (IIM); thus, dysregulated miRNA expression may be involved in disease pathogenesis (8–11). Besides, the expression of inflammatory miRNAs and anti-troponin-targeted miRNAs is increased in mice with severe myositis, indicating that miRNAs are involved in muscle injury, inflammation, and myasthenia (12). Our previous study also revealed that several immune-related miRNAs are correlated with dermatomyositis (13); however, the involvement of miRNAs in IMNM remains unclear.

RNA-binding proteins (RBPs) are known to change the fate or function of bound RNA. Of such RBPs, HuR, a member of the ELAVL1/Hu family, has been reported to modulate the fusion of myoblasts into myotubes during skeletal myogenesis by coordinating the expression of myogenic regulatory factors such as myogenin (MyoG), MyoD, and p21 (14–16). Nevertheless, the role of HuR in IMNM has not been investigated.

In this study, we aimed to explore the role and mechanism of abnormally expressed miR-18a-3p and its target RBP HuR in the myogenesis of patients with IMNM. Our results may help in better understanding the pathogenesis of IMNM and developing effective therapeutic targets.

## Materials and Methods

### Study Population

Patients with IMNM, DM and healthy controls (HCs), who participated in this study, were admitted to the Department of Rheumatology and Physical Examination from 2017 to 2019 in China–Japan Friendship Hospital, Beijing, China. The age and gender of the patients with IMNM were matched with those of the HCs. IMNM was classified according to the pathological criteria described at the 224th European Neuromuscular Center International Workshop (17). This study did not include patients with other autoimmune diseases, tumors, and infections. Skeletal muscle tissue biopsies were obtained from 18 patients with IMNM, 11 patients with DM and 8 HCs. The study was approved by the Ethical Review Committee of the China–Japan Friendship Hospital (2019-25-K19). Before commencing the study, we obtained written informed consent from all patients for the use of their tissues and data.

### Clinical Assessment

Detailed results of physical examinations and routine laboratory tests of all patients were recorded at their first visit. Routine laboratory parameters, including the levels of serum C-reactive protein, creatine kinase, alanine aminotransferase, aspartate aminotransferase, and lactate dehydrogenase, and erythrocyte sedimentation rate, were assessed. The damage to the patients with IMNM was evaluated using manual muscle testing 8 (MMT8). A 10 cm visual analog scale (VAS) was used to evaluate the muscle activity component of the myositis disease activity assessment tool (MDAAT-muscle) and physician global assessment (PGA) for disease activity (18).

Serum anti-HMGCR antibodies were detected using an enzyme-linked immunosorbent assay (ELISA) kit (Inova Diagnostics, San Diego, CA, USA). Serum anti-SRP antibodies were detected using a commercially available kit (Euroimmun, Lübeck, Germany).

### Myoblast Culture and Differentiation

Human skeletal muscle myoblasts (HSMM) were obtained from Lonza Bioscience (Basel, Switzerland) and cultured using the Clonetics skeletal muscle myoblast cell system (Lonza) in a humidified incubator (Thermo Fisher Scientific, Waltham, MA, USA) at 37°C with 5% CO_2_. The cells were seeded in 12-well or 6-well plates and incubated for 18 h before transfection. At 80–90% confluence, the cells were transferred into Dulbecco’s modified Eagle medium supplemented with 2% horse serum and 1% penicillin-streptomycin for inducing differentiation.

### Cell Transfection

HSMM were seeded in 12-well or 6-well plates; when the cells reached approximately 70% confluence, they were transfected with 100 nM of small interfering RNA targeting HuR (siHuR) or small interfering control RNA (siCtrl), miR-18a-3p mimics or mimics negative control (NC) and miR-18a-3p inhibitor or inhibitor NC (Ribobio, Guangzhou, China) using Lipofectamine 3000 (Invitrogen, Carlsbad, CA, USA) according to the manufacturer’s instructions. The culture system was replaced with fresh medium at 12 h post-transfection. The cells were used to stimulate differentiation at 24 h post-transfection.

### Dual-Luciferase Reporter Gene Assay

Dual-luciferase reporter gene assay was used to confirm the binding sites of miR-18a-3p and the 3′ untranslated region (UTR) of HuR mRNA. A pmirGLO-HuR-wild type (Hur-WT) or a pmirG-LO-HuR-mutant type (HuR-MUT) and miR-18a-3p mimics or mimics NC were co-transfected into HEK293T cells using Lipofectamine 2000 (Invitrogen). Dual-Glo luciferase assay system (Promega, Madison, WI, USA) was used to measure the luciferase activity in the cell lysates according to the manufacturer’s instructions. The ratios of Firefly to Renilla luciferase activities were used as the result of the dual-luciferase reporter gene assay. The experiments were performed in three independent replicates.

### Bioinformatics Analysis

The online databases miRwalk (http://mirwalk.umm.uni-heidelberg.de/) and TargetScan (http://www.targetscan.org/vert_72/) were used to search for the targets of miR-18a-3p (19, 20). Venny (https://bioinfogp.cnb.csic.es/tools/venny/index.html) was used to obtain the common targets from above mentioned databases. Heatmap software(ggplot2) was applied to cluster the enrichment terms obtained after Kyoto Encyclopedia of Genes and Genomes-based analysis of the common genes.

### RNA Extraction and Quantitative Reverse-Transcription Polymerase Chain Reaction

Trizol (Invitrogen) was used to extract total RNA from the muscle tissues according to the manufacturer**’**s instructions. PrimeScript™ RT reagent Kit (Takara, Otsu, Japan) was used to reverse transcribe RNA into complementary DNA (cDNA). Quantitative reverse-transcription PCR (qRT-PCR) was performed using an ABI 7500 sequence detection system (Applied Biosystems, Foster City, CA) with SYBR Green Master Mix (Qiagen, Hilden, Germany). The thermal conditions were as follows: 95**°**C for 2 min, followed by 40 cycles at 95**°**C for 5 s and 60**°**C for 30 s. *GAPDH* was used as the internal reference of gene expression. Each sample was measured in triplicate. The U6 small RNA was used as the internal reference for estimating the levels of miR-18a-3p and other members in miR-17-92 cluster. All relevant PCR primers used in this study are listed in **Supplementary Table 1**.

### Western Blotting Analysis

NE-PER Nuclear and Cytoplasmic Extraction Reagents kit (Thermo Fisher Scientific) was used to extract nuclear-cytoplasmic fraction according to the manufacturer’s instructions. NP-40 lysis buffer (Beyotime, Shanghai, China) and phenylmethylsulfonyl fluoride (Solarbio, Beijing, China) were used to obtain total protein. Protein quantification were performed by BCA Protein Assay kit (Thermo Fisher Scientific) as per the manufacturer’s instructions. The available protein was denatured with 5× sodium dodecyl sulfate (SDS)-PAGE denatured protein loading buffer (Applygen, Beijing, China) at 98°C for 5 min. Next, SDS-polyacrylamide discontinuous gel electrophoresis was used to separate the denatured protein; then, the protein was transferred to nitrocellulose filter membranes (Millipore, Boston, MA, USA) by wetting transfer method for 90 min. Immunoblotting was conducted using specific antibodies listed in **Supplementary Table 2** and horseradish peroxidase-conjugated secondary antibody. The membranes were visualized using ChemiDoc XRS+ System (Bio-Rad, Hercules, CA, USA), and the grayscale value of the proteins was estimated using Image Lab 6.0.1.

### Immunofluorescence Staining

Differentiated HSMM myotubes or primary myotubes were fixed in 95% ethanol. Then, 0.3% Triton X-100 was used for 15 min for cell membrane penetration. The samples were washed three times, for 5 minutes each time, and then blocked with goat serum for 2 h. The samples were then incubated with anti-HuR (1:500; Abcam, Cambridge, MA, USA), anti-MyoG (1:250; Abcam), and anti-myosin heavy chain (MyHC; 1:400; Santa Cruz Biotechnology, Dallas, TX, USA) overnight at 4°C, and then with Alexa 488-conjugated or Alexa 555-conjugated secondary antibody (Abcam) for 30 min at 25°C. Next, 4′,6-diamidino-2-phenylindole (DAPI; Beyotime) was used to stain the cell nuclei, which were observed under a fluorescence microscope (Olympus, Tokyo, Japan). The degree of differentiation was measured by a fusion index, which is defined as the number of nuclei in the myotube and as a percentage of the total nuclei.

### Statistical Analysis

Continuous data were expressed as means ± standard deviation for normal distribution or as median and interquartile range for those without normal distribution. Student’s *t*‐test was used for separating the means of the normal data, whereas Mann–Whitney U test for data that deviated from normal. Spearman’s rank-order correlation was used to test correlations of data with non-normal distribution. A *p* value < 0.05 was considered as statistically significant. SPSS 22.0 (IBM, Armonk, NY, USA) and GraphPad Prism 8.0 (GraphPad Software, San Diego, CA, USA) were used for all statistical analyses.

## Results

### Clinical Characteristics of Patients With IMNM

The clinical characteristics of the patients with IMNM are presented in **Table 1**. Of the 18 patients with IMNM, 61.1% were female. The average disease onset age was 47.6 years, and the median disease duration was 5 months. 12 patients were positive to the anti-SRP-autoantibody, 2 to the anti-HMGCR-autoantibody, and the remaining four patients were myositis-specific autoantibody negative. The average VAS score was 4.7, and the average MMT8 score was 58.0.

**Table 1:** Clinical features of immune-mediated necrotizing myopathy (IMNM) patients.

Characteristics	Patients (*n* = 18)	HCs (*n* = 8)
Female, no. (%)	11 (61.1%)	5 (62.5%)
Age of onset, mean ± s.d. years	47.6± 15.6	50.8 ± 17.1
Disease duration, median of IQR months	5(2-9.7)	
Clinical features, no. (%)		
Muscle weakness	15 (83.3%)	
Myalgia	6 (33.3%)	
Laboratory data		
MSA, no. (%)		
Anti-SRP- positive	12 (66.6%)	
Anti-HMGCR- positive	2 (11.1%)	
MSA-negative	4 (22.2%)	
CK (IU/L), median (IQR)	3597 (1356-7521)	
ALT (IU/L), median (IQR)	207.5 (61.4-319)	
AST (IU/L), median (IQR)	119.5 (39.7-160.8)	
LDH (IU/L), median (IQR)	674 (426.5-1113)	
ESR (mm/h), median (IQR)	7 (5-20.5)	
CRP (mg/dl), median (IQR)	0.36 (0.25-0.45)	
MMT8, mean ± s.d.	58.0 ± 12.9	
PGA VAS, mean ± s.d.	4.7 ± 1.8	

Average values or numbers of each group are shown. Standard deviation (s.d.), interquartile range (IQR), or percentages are shown. MSA, myositis-specific antibody; IMNM, immune-mediated necrotizing myopathy; SRP, signal recognition particle; HMGCR, 3-hydroxy-3-methyl coenzyme A reductase; CK, creatine kinase; ALT, alanine aminotransferase; AST, aspartate aminotransferase; LDH, lactate dehydrogenase; ESR, erythrocyte sedimentation rate; CRP, C-reactive protein; PGA, physician global assessment; VAS, visual analog scale; MMT8, manual muscle test 8 (0-80).

### miR-18a-3p Expression in Skeletal Muscles of Patients With IMNM

**Figure 1:**
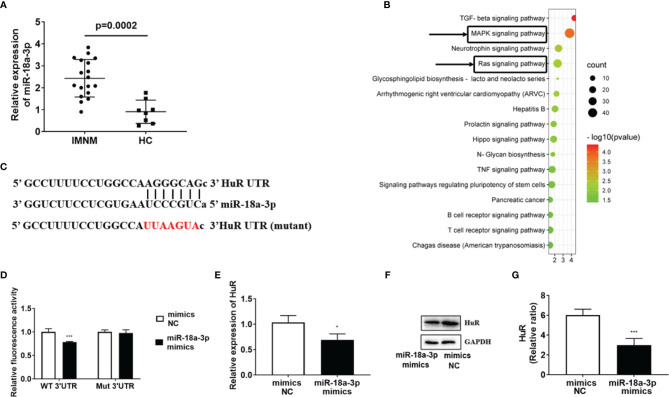
miR-18a-3p is highly expressed in skeletal muscle of patients with immune-mediated necrotizing myopathy (IMNM). **(A)** miR-18a-3p levels in patients with IMNM and healthy controls (HCs) as determined by quantitative reverse-transcription polymerase chain reaction PCR (qRT-PCR). **(B)** Kyoto Encyclopedia of Genes and Genomes (KEGG) pathway analysis of miR-18a-3p target genes. **(C)** Sequence alignment of the 3’ untranslated region (UTR) of HuR mRNA with miR-18a-3p. Nucleotides in deletion mutant (Mut) HuR binding sites are marked in red. **(D)** Effects of miR-18a-3p on the activity of luciferase reporters, bearing wild-type (WT) or Mut on the binding sites of the 3′ UTR of HuR mRNA. **(E)** HuR levels at 24 h post-transfection with miR-18a-3p mimics as determined by qRT-PCR. **(F)** Protein levels of HuR at 24 h post-transfection with miR-18a-3p mimics as determined by western blot analysis. **(G)** Glyceraldehyde 3-phosphate dehydrogenase (GAPDH) was used as an internal control for western blot analysis to normalize the relative protein expression. Relative mRNA expression was estimated using the 2-^ΔΔCt^ method. Data are expressed as means ± standard deviation (SD). *, *** indicate significant differences of p < 0.05 and p < 0.001, respectively. miR, microRNA; NC, negative control.

We found that the expression level of miR-18a-3p was significantly higher in patients with IMNM than in the HCs (p=0.0002) (**Figure 1A**). Since miR-18a-3p is the member of miR-17-92 cluster, and the miR-17-92 cluster participates in skeletal muscle differentiation (7), we further detected the expression of other miRNAs in this cluster in these samples (**Supplementary Figures 1A**–**E**). The Kyoto Encyclopedia of Genes and Genomes pathway enrichment analysis revealed that the target genes of miR-18a-3p were involved in regulating numerous pathways, ras and mitogen-activated protein kinase (MAPK) signaling pathway are involved in the regulation of myogenic differentiation (21) (**Figure 1B**). Bioinformatics analysis predicted that HuR may be a target gene of miR-18a-3p (**Figure 1C**). Dual-luciferase reporter gene assay showed that overexpression of miR-18a-3p decreased the luciferase activity of the plasmid containing HuR-WT, that is, the luciferase activity of miR-18a-3p binding sequence; however, it did not decrease the activity of HuR-MUT (**Figure 1D**). Furthermore, overexpression of miR-18a-3p strongly suppressed HuR transcription at 24 h post-transfection (**Figure 1E**). Accordingly, western blot analysis showed that the protein levels of HuR were reduced by the transfection of the miR-18a-3p mimics (**Figures 1F, G**). These results demonstrated that miR-18a-3p may directly interact with the predicted target site in HuR.

### HuR Expression Levels in Patients With IMNM

**Figure 2:**
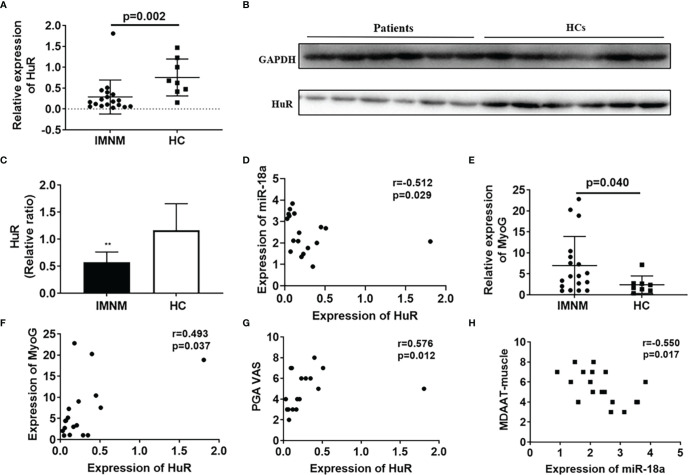
The mRNA and protein levels of HuR are decreased in skeletal muscles of patients with immune-mediated necrotizing myopathy (IMNM). **(A)** mRNA levels of HuR in patients with IMNM and healthy controls (HCs) as determined by qRT-PCR. **(B)** Protein levels of HuR in patients with IMNM and HCs as determined by western blot analysis. **(C)** GAPDH was used as an internal control for western blot analysis to normalize the relative protein expression. **(D)** Correlation of HuR and miR-18a-3p mRNA levels in patients with IMNM. **(E)** mRNA levels of myogenin (MyoG) in skeletal muscles of patients with IMNM and HCs as determined by qRT-PCR. **(F)** Correlation of HuR and MyoG mRNA levels in patients with IMNM. **(G)** Correlation of HuR mRNA levels and Patient Global assessment of disease activity by VAS (0-10). **(H)** Correlation of miR-18a-3p levels and the muscle activity component of the Myositis Disease Activity Assessment Tool by VAS (MDAAT-muscle). Relative mRNA expression was estimated using the 2^-ΔΔCt^ method. Data are expressed as means ± standard deviation (SD). ** indicate significant differences of p < 0.01.

Our results showed that the expression level of HuR was significantly reduced in patients with IMNM than in HCs (p=0.002) (**Figure 2A**), but the expression of HuR in DM patients is significantly higher than in HCs (**Supplementary Figure 2**). Western blot analysis revealed a decrease in the protein levels of HuR in patients with IMNM than in the HCs (**Figures 2B, C**). At the same time, miR-18a-3p expressed in patients with IMNM were negatively correlated with those of HuR (r=-0.512, p = 0.029; **Figures 2D**). Furthermore, MyoG was highly expressed in patients with IMNM, and its transcription levels were positively correlated with those of HuR (*r* = 0.493, *p* = 0.037; **Figures 2E, F**). Besides, the VAS score of patients with IMNM was positively correlated with HuR levels (*r* = 0.576, *p* = 0.012); however, the muscle activity component of the Myositis Disease Activity Assessment Tool by VAS (MDAAT-muscle) was negatively correlated with miR-18a-3p expression (*r* = -0.550, *p* = 0.017; **Figures 2G, H**). No association was identified between HuR or miR-18a-3p expression and the MMT8 score.

### Involvement of HuR in Myoblast Differentiation

**Figure 3:**
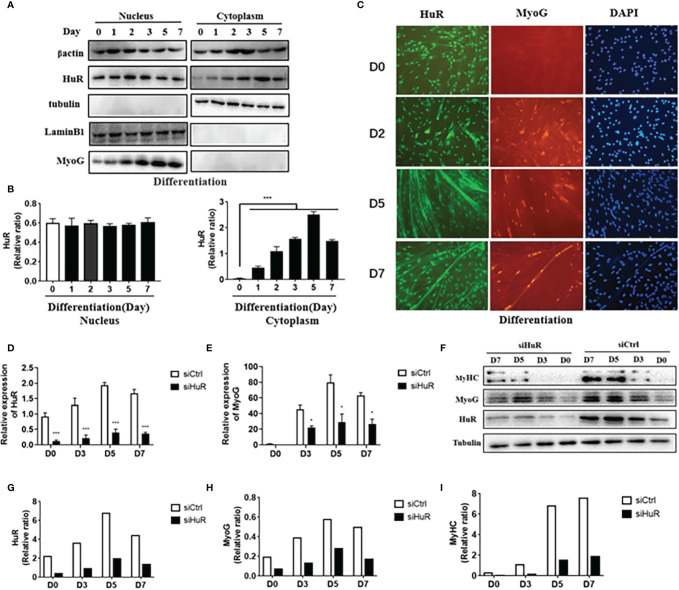
Expression of HuR in human skeletal muscle myoblasts (HSMM) during differentiation. **(A)** Nuclear and cytoplasmic HuR and myogenin (MyoG) protein levels in HSMM during differentiation as determined by western blot analysis. **(B)** HuR levels in the human myoblast nucleus as normalized with lamin-B1 (left) and in the human myoblast cytoplasm as normalized with tubulin (right). **(C)** Immunofluorescence detection of HuR (green) and MyoG (red) in HSMM during differentiation. DAPI (blue) is used as a nuclear stain. Scale bar, 100 μm. **(D)** mRNA levels of HuR post-transfection with small interfering RNA targeting HuR (siHuR) at days 0, 3, 5, and 7 of differentiation as determined by qRT-PCR. **(E)** mRNA levels of MyoG post-transfection with siHuR at days 0, 3, 5, and 7 of differentiation as determined by qRT-PCR. **(F)** Western blot analysis was used to determine HuR, MyoG, and MyHC protein levels post-transfection with siHuR at days 0, 3, 5, and 7 of differentiation. **(G–I)** GAPDH was used as an internal control for western blot analysis to normalize relative protein expression. Relative mRNA expression was estimated using the 2^-ΔΔCt^ method. Data are expressed as means ± standard deviation (SD). *, *** indicate significant differences of p < 0.05 and p < 0.001, respectively.

As shown in **Figures 3A, B**, the nuclear abundance of HuR did not change noticeably during differentiation, whereas the cytoplasmatic abundance increased remarkably at the onset of differentiation and remained elevated throughout the process. We observed that the expression levels of MyoG were consistent with those of cytoplasmic HuR; these results were confirmed using immunofluorescence staining of human myoblasts after differentiation (**Figure 3C**). Next, we use siRNA technology to solve the role of HuR in myoblast. Three different siRNAs were designed to target human HuR and transfected in myoblast. The results showed that siHuR effectively down-regulated the mRNA expression in myoblast (**Supplementary Figures 3A**). According to qRT-PCR analysis, transfection of siHuR (100 nM) significantly reduced the cellular level of HuR (>70%) (**Supplementary Figures 3B**). Besides, we observed that transfection of siHuR significantly inhibited the expression levels of HuR and MyoG during differentiation (**Figures 3D, E**). Western blot analysis showed that the protein levels of HuR, MyoG, and MyHC in siHuR-treated myoblasts were significantly decreased when compared with that in the control (**Figures 3F**–**I**). These results suggested that HuR knockdown could inhibit myoblast differentiation.

### HuR Regulation by miR-18a-3p in Myogenic Differentiation

**Figure 4:**
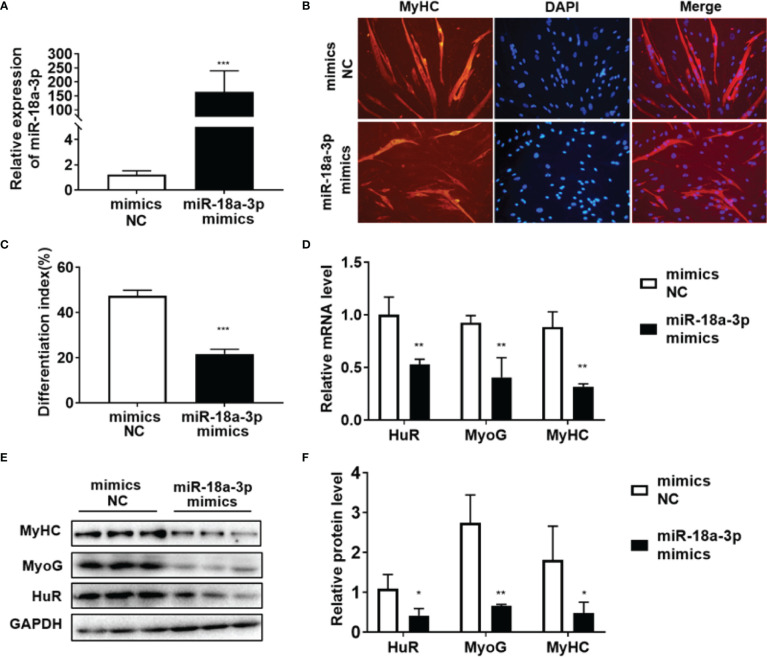
Overexpression of miR-18a-3p inhibits myogenic differentiation. **(A)** miR-18a-3p levels post-transfection with miR-18a-3p mimics at day 5 of differentiation as determined by qRT-PCR. **(B)** Immunofluorescence detection of MyHC (red) in human myoblasts. Scale bar, 100 μm. **(C)** Fusion index (ratio of MyHC positive myotubes with ≥ 2 nuclei to the total number of nuclei). **(D)** mRNA levels of HuR, MyoG, and MyHC post-transfection with miR-18a-3p mimics at day 5 of differentiation as determined by qRT-PCR. **(E, F)** HuR, MyoG, and MyHC protein levels post-transfection with miR-18a-3p mimics at day 5 of differentiation as determined by western blot analysis. Relative mRNA expression was estimated using the 2-^ΔΔCt^ method. Data are expressed as means ± standard deviation (SD). *, **, *** indicate significant differences of p < 0.05, p < 0.01, and p < 0.001, respectively. NC, negative control.

**Figure 5:**
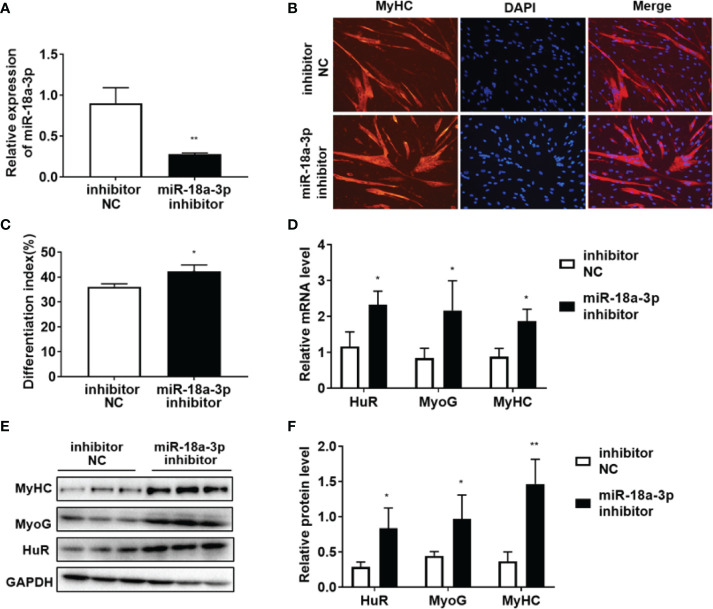
Inhibition of miR-18a-3p promotes myogenic differentiation. **(A)** miR-18a-3p levels post-transfection with miR-18a-3p inhibitor at day 5 of differentiation as determined by qRT-PCR. **(B)** Immunofluorescence detection of MyHC (red) in human myoblasts. Scale bar, 100 μm. **(C)** Fusion index (ratio of MyHC-positive myotubes with ≥ 2 nuclei to the total number of nuclei). **(D)** mRNA levels of HuR, MyoG, and MyHC post-transfection with miR-18a-3p inhibitor at day 5 of differentiation as determined by qRT-PCR. **(E, F)** HuR, MyoG, and MyHC protein levels post-transfection with miR-18a-3p inhibitor at day 5 of differentiation as determined by western blot analysis. Relative mRNA expression was estimated using the 2-^ΔΔCt^ method. Data are expressed as means ± standard deviation (SD). *, ** indicate significant differences of p < 0.05 and p < 0.01, respectively. NC, negative control.

As shown in **Figure 4A**, miR-18a-3p mimics transfection significantly increased miR-18a-3p expression. MyHC immunofluorescence staining on day 5 of differentiation showed that overexpression of miR-18a-3p reduced the fusion rate of myoblasts in comparison to that of the mimics NC (**Figures 4B, C**). Furthermore, miR-18a-3p mimics transfection significantly suppressed the transcription and protein levels of HuR, MyoG, and MyHC (**Figures 4D**–**F**). The opposite results were obtained after transfection of the miR-18a-3p inhibitor (**Figures 5A**–**F**). Our data demonstrated that inhibition of miR-18a-3p might promote myogenic differentiation by targeting HuR.

## Discussion

IMNM is a systemic disease characterized by chronic inflammation in muscle tissues, causing weakness and myofiber necrosis (1). In the present study, we explored the role of differentially expressed miRNAs and target genes in regulating the regeneration and differentiation of muscle tissues in patients with IMNM.

miRNA is involved in the regulation of a wide range of developmental and physiological cell processes, including differentiation, proliferation, growth, and apoptosis (22). Previous studies have shown that miRNAs are abnormally expressed in the muscles of patients with IIM and are probably involved in disease pathogenesis. For instance, miR-221, miR-155, miR-146b, miR-214, and miR-222 are upregulated in 10 primary muscle diseases, including IIM (23). Furthermore, miRNAs found in skeletal muscles and myocardium, such as miR-1, miR-133a/b, miR-208, miR-486, miR-206, and miR-499, participate in the myogenic process by modulating the expression of myogenic regulatory factors (24). Of these, the expression of miR-1 and miR-133a/b is decreased in the muscle tissues of patients with IIM and is negatively correlated with that of TNF-α. The latter inhibits the expression of myogenic miRNAs in an NF-κB-dependent manner, suppressing the differentiation of C2C12 myoblasts into myotubes and leading to muscle regeneration (25). In our study, miR-18a-3p was upregulated in patients with IMNM and participated in myogenic differentiation by regulating HuR expression. We also found that miR-18a-3p expression was negatively related to the VAS score, revealing the potential role of this miRNA in muscle inflammation and the degenerative pathology of IMNM. A previous study on mice with severe myositis has shown that increased expression of inflammatory miRNAs (miR-146a, miR-142-3p, miR-142-5p, and miR-455-5p) and targeted muscle dystrophins (miR-146a, miR-146b, miR-31, and miR-223) are regulated by NF-κB, and may be related to muscle injury, inflammation, and myasthenia (12). Of note, in our study, we found that the expression of HuR in IMNM was decreased compared to HC, while the expression of HuR in the muscle tissue of DM patients was significantly higher than in the HC. The results of this study further indicate that the muscle damage mechanism of DM may be different from that of IMNM.

The miR-17-92 cluster participates in normal development, skeletal myogenesis, immune disease, cardiovascular disease, and tumorigenesis (26, 27), of which miR-20a, miR-92, and miR-17 regulate the proliferation of myoblasts but inhibit their differentiation by targeting ENH1. Further, miR-17 and miR-20a effectively promote the differentiation of C2C12 cells; whereas miR-18a delays the differentiation of the same cells (7, 28). Here, we use bioinformatics software and luciferase reporter gene detection to show that HuR, a RNA-binding protein, is the direct target of miR-18a-3p. Under the overexpression miR-18a-3p, the expression level of HuR RNA and protein was significantly inhibited, and further inhibit myogenic differentiation. On the contrary, interference of miR-18a-3p promoted the expression of HuR and further promote myogenic differentiation. In our study, we found that expression of miR-18a-3p in patients with IMNM was negatively correlated with HuR. miR-18a-3p affected myoblast differentiation by downregulating the expression of HuR. In contrast, use of an miR-18a-3p inhibitor led to opposite results.

HuR is an RBP that protects the 3’-UTR of mRNA from decay and degradation by stabilizing the adenylate-uridylate-rich elements. HuR is mainly present in the nucleus, where it combines with the target mRNA, to form a complex that moves to the cytoplasm. When HuR dissociates from the target mRNA, it returns to the nucleus (29). Previous studies have revealed that HuR regulates various myogenic factors, including MyoG, MyoD, and p21, and that its cytoplasmic accumulation is essential in myogenesis (16, 30). In the present study, we found that the expression of cytoplasmic HuR in myoblasts increased as differentiation progressed and was consistent with that of MyoG. Transfection of siHuR into myoblasts decreased the mRNA and protein levels of MyoG and MyHC in the differentiated myoblasts, thereby inhibiting cell differentiation.

HuR is also involved in the pathogenesis of inflammatory diseases (e.g., rheumatoid arthritis) by binding to the mRNAs of inflammatory cytokines (e.g., TNF-α) (31, 32). We found that the expression of HuR was positively correlated with disease activity in patients with IMNM, suggesting that the participation of this protein in inflammatory response is by stabilizing the mRNAs of inflammatory factors; however, further research is needed to confirm these results.

**Figure 6:**
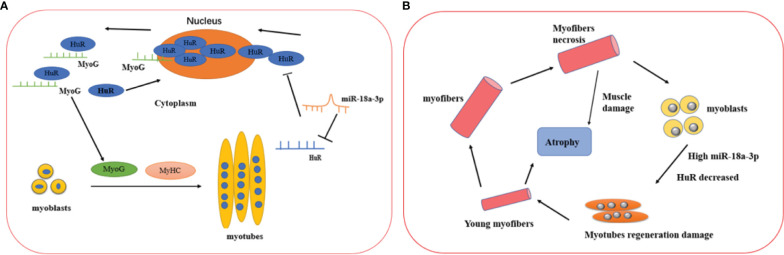
Proposed mechanisms of miR-18a-3p and its target HuR in the IMNM pathogenesis and healthy controls. **(A)** The physical role of miR-18a-3p and HuR in the myogenesis of skeletal muscle. miR-18a-3p is mainly involved in the post-transcriptional regulation of HuR to inhibit HuR expression. HuR is abundantly localized to the cytoplasm in differentiating myoblasts, which promotes the fusion of myoblasts to form myotubes by stabilizing several myogenic regulatory factor mRNAs such as MyoG. After the myogenesis, HuR returns into the nucleus in the differentiated myotubes. **(B)** The proposed mechanisms of miR-18a-3p and HuR in the IMNM pathogenesis. Skeletal muscle was damaged and necrosis, which lead to highly expressed miR-18a-3p and decreased HuR, then myoblast regeneration was impaired by the complex interaction of miR-18a-3p and HuR. Ultimately, the skeletal muscle prompt to atrophy.

Overall, our study revealed that miR-18a-3p was significantly upregulated, whereas its target gene HuR was downregulated in the muscle tissues of patients with IMNM compared to DM and HCs. miR-18a-3p were negatively correlated with muscle activity in patients with IMNM, but the expression levels of HuR were positively correlated with disease activity in patients with IMNM. Finally, miR-18a-3p and HuR might regulate myoblast differentiation (**Figure 6**). To the best of our knowledge, this is the first study to report the mechanism of miR-18a-3p inhibiting myogenic differentiation in IMNM.

## Data Availability Statement

The data presented in the study are available from the corresponding authors upon request.

## Ethics Statement

The studies involving human participants were reviewed and approved by Ethical Review Committee of the China–Japan Friendship Hospital. The patients/participants provided their written informed consent to participate in this study. Written informed consent was obtained from the individual(s) for the publication of any potentially identifiable images or data included in this article.

## Author Contributions

LY, YZ, GW, and XS participated in the conception and design of the experiments. LY and YZ performed the experiments and data analysis. LY, YZ, and XS wrote the manuscript. FC, QP, XL, and GW supervised the manuscript. All authors contributed to the article and approved the submitted version.

## Funding

This work was supported by the Elite Medical Professionals project of China-Japan Friendship Hospital (NO.ZRJY2021-GG14), the Youth Program of the National Natural Science Foundation of China (grant numbers 81401363, 81601367), Major Research Plan of the National Natural Science of China(grant number 91542121), National Natural Science Foundation of China(grant number 81971531),Capital Health Research and Development of Special Programs (grant number 2014–4-4062), and The Fundamental Research Funds for the Central Universities (grant number 3332020074).

## Conflict of Interest

The authors declare that the research was conducted in the absence of any commercial or financial relationships that could be construed as a potential conflict of interest.

## Publisher’s Note

All claims expressed in this article are solely those of the authors and do not necessarily represent those of their affiliated organizations, or those of the publisher, the editors and the reviewers. Any product that may be evaluated in this article, or claim that may be made by its manufacturer, is not guaranteed or endorsed by the publisher.

## Abbreviations

IMNM, immune-mediated necrotizing myopathy; UTR, untranslated region; HSMM, human skeletal muscle myoblasts; ELISA, enzyme-linked immunosorbent assay; PGA, physician global assessment; MMT8, manual muscle testing 8; HC, healthy controls; MyoG, myogenin; IIM, idiopathic inflammatory myopathy; RBP, RNA-binding protein; HMGCR, anti-3-hydroxy-3-methylglutaryl-CoA reductase; anti-SRP, anti-signal recognition particle; qRT-PCR, quantitative reverse-transcription real-time polymerase chain reaction.
