# Supplementary material for: miR-18a-3p and Its Target Protein HuR May Regulate Myogenic Differentiation in Immune-Mediated Necrotizing Myopathy
Source: Front Immunol. 2022 Jan 5;12:780237. doi: 10.3389/fimmu.2021.780237 (PMC8766969; doi:10.3389/fimmu.2021.780237)
Supplement: Supplementary file 1 [file DataSheet_1.pdf]

Supplementary Material

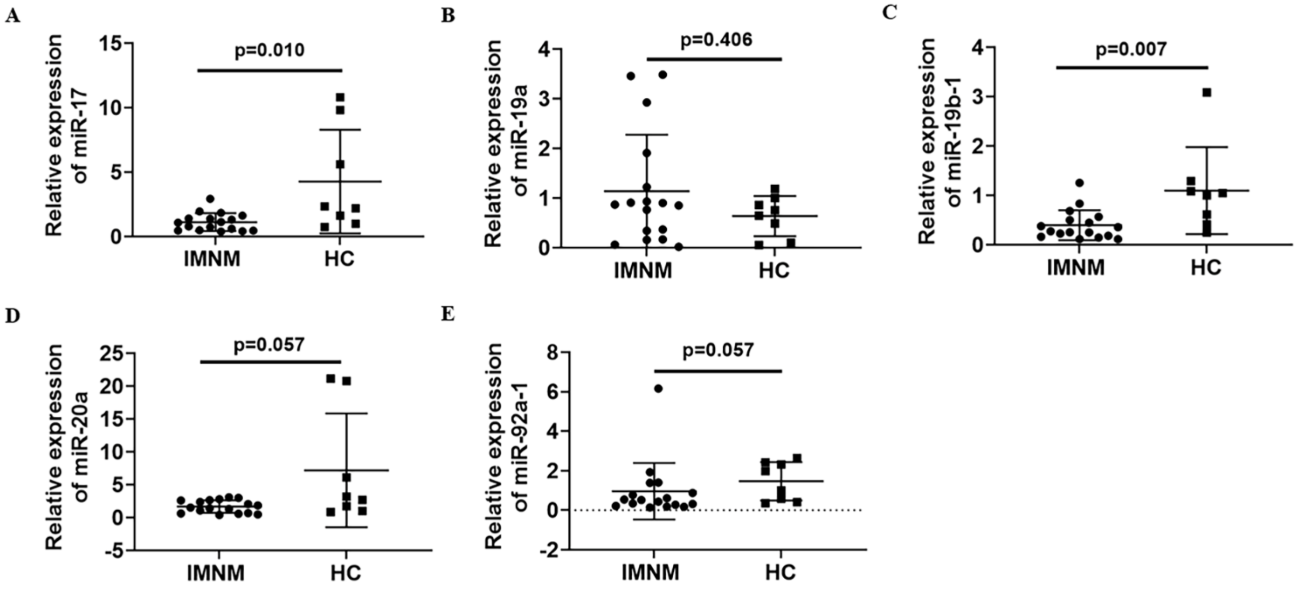

Supplementary Figure 1. The miR-17-92 cluster is differentially expressed in the skeletal muscle of patients with immune-mediated necrotizing myopathy (IMNM). (A) miR-17 levels in patients with IMNM and healthy controls (HCs) as determined by quantitative reverse-transcription polymerase chain reaction PCR (qRT-PCR). **(B)** miR-19a levels in patients with IMNM and HCs as determined by qRT-PCR. **(C)** miR-19b-1 levels in patients with IMNM and HCs as determined by qRT-PCR. **(D)** miR-20a levels in patients with IMNM and HCs as determined by qRT-PCR. **(E)** miR-92a-1 levels in patients with IMNM and HCs as determined by qRT-PCR. Relative mRNA expression was estimated using the $2^{-\Delta\Delta Ct}$ method. Data are expressed as means ± standard deviation (SD). miR, microRNA.

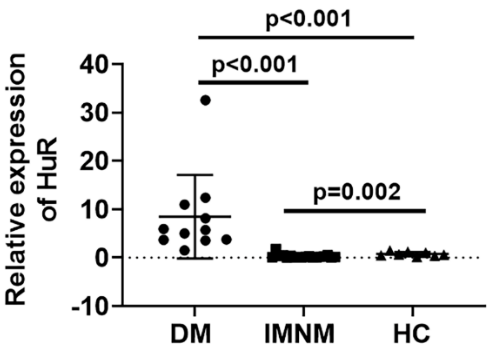

Supplementary Figure 2. The mRNA levels of HuR are decreased in skeletal muscles of patients with immune-mediated necrotizing myopathy (IMNM) compared to HCs, but the

expression of HuR in DM patients is significantly higher than in HCs.

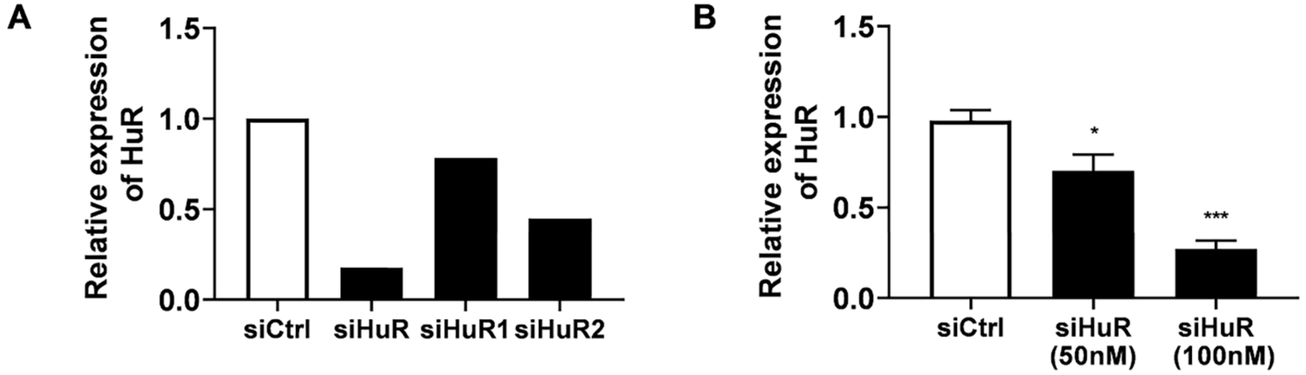

Supplementary Figure 3. Knock-down of HuR by RNA interference (siRNA) in myoblast. (A) Three different siRNAs using siHuR decreases expression of HuR at mRNA levels compared with the control group(siCtrl) by quantitative reverse-transcription polymerase chain reaction PCR (qRT-PCR). **(B)** HuR mRNA levels under different siHuR concentrations are determined by qRT-PCR. Relative mRNA expression was estimated using the $2^{-\Delta\Delta Ct}$ method. Data are expressed as means ± standard deviation (SD). *, *** indicate significant differences of $p < 0.05$ and $p < 0.001$, respectively. miR, microRNA.

Supplementary Table 1. Primer sequences used for real-time PCR analysis of gene expression

Gene	Primer sequence	
miR-18a-3p	Stem-loop primer	CTCAACTGGTGTCGTGGAGTCGGCAATT CAGTTGAGCTATCTGC
	Sense	TCGCCTAAGGTGCATCTAGTGC
	Forward	CTCAACTGGTGTCGTGGAGTCGGC
U6	Sense	CTCGCTTCGGCAGCACA
	Forward	AACGCTTCACGAATTTGCGT
GAPDH	Sense	GGAGCGAGATCCCTCCAAAAT
	Forward	GGCTGTTGTCATACTTCTCATGG
HuR	Sense	CAGCATTGGTGAAGTTGAATCTG
	Forward	CCTTAATGGTTTTTGACTGGAGC
MyoG	Sense	AAGTGAATGAGGCCTTCGAG
	Forward	AGATTGTGGGCGTCTGTAGG
MyHC	Sense	CCCAGATGAAGGAGCTATGG
	Forward	CTTTTCACTTTCCCGATTCTGG
miR-17	Forward	GGCAAAGTGCTTACAGTGCAGGTAG
miR-19a	Forward	CGCCTGTGCAAATCTATGCAAAACTGA

miR-19b-1	Forward	AGTTTTGCAGGTTTGCATCCAGC
miR-20a	Forward	CGCGTAAAGTGCTTATAGTGCAGGTAG
miR-92a-1	Forward	CAGGTTGGGATCGGTTGCAATG

Supplementary Table 2. List of antibodies used for western blot

Antibody	Manufacturer	Cat. No.
HuR	Abcam, Cambridge, MA, USA	ab200342
MyoG	Abcam, Cambridge, MA, USA	Ab1835
MyHC	Santa Cruz Biotechnology, Dallas, TX, USA	sc-376175
GAPDH	Abcam, Cambridge, MA, USA	ab8245
β-actin	Abcam, Cambridge, MA, USA	ab8226
Tubulin	Proteintech, Wuhan, China	10068-1-AP
LaminB1	Abcam, Cambridge, MA, USA	ab133741
